# Posterior Scleritis: A Case Report and Literature Review of the Management of the Condition

**DOI:** 10.7759/cureus.61537

**Published:** 2024-06-02

**Authors:** Raheem Robertson, Fawwad A Ansari, Stefan Gafoor, Osahon N Idolor, Dominic Amakye

**Affiliations:** 1 Graduate Medical Education, Piedmont Athens Regional Hospital, Athens, USA

**Keywords:** rheumatoid arthritis, systemic lupus erythematosus, anca-associated vasculitis, scleritis, lupus, inflammation of eye, biologic therapies, ocular manifestations, internal medicine and rheumatology

## Abstract

Posterior scleritis is a rare inflammatory eye condition affecting the posterior segments of the sclera and is more prevalent in females. Its clinical presentation, often nonspecific, includes ocular pain, headache, and vision loss. Misdiagnosis is common due to a lack of specific symptoms posing a potential threat to vision. The etiology is often tied to rheumatic diseases, such as rheumatoid arthritis (RA), systemic erythematous lupus (SLE), and anti-neutrophil cytoplasmic antibodies (ANCA)-associated vasculitis. Posterior scleritis poses diagnostic challenges, mimicking many other ocular conditions, hence necessitating a thorough clinical eye exam. Laboratory studies, including inflammatory markers and markers of rheumatic diseases, may identify underlying systemic diseases. Imaging, including B-scan ultrasound and magnetic resonance imaging (MRI), aids in accurate diagnosis. Treatment involves non-steroidal anti-inflammatory drugs (NSAID), as well as topical corticosteroids for mild disease and systemic corticosteroids for severe disease. Biologic therapy has become increasingly significant for refractory cases. A multidisciplinary approach involving ophthalmology and rheumatology is crucial in the management of this potential sight-threatening disease. This case report highlights a 46-year-old woman with a history of RA-associated posterior scleritis.

## Introduction

Posterior scleritis is a rare inflammatory condition of the eye, involving the posterior segment of the sclera [1]. Posterior scleritis is more common in females than males, with a mean age of onset in the 40s, and is the rarest form of scleritis ranging between 3% and 17% of all cases [1-3].

Clinical manifestations are very nonspecific and are usually monocular. About one-third of cases are binocular, making bilateral disease even rarer [4]. Symptoms usually include ocular pain, which is exacerbated by ocular movements, headache, and loss of vision [1]. It is often misdiagnosed and potentially sight-threatening because of its non-specific presentation; a high index of suspicion and expertise are needed for diagnosis [2]. Posterior scleritis is commonly associated with rheumatic diseases, such as rheumatoid arthritis (RA), anti-neutrophil cytoplasmic antibodies (ANCA)-associated vasculitis, and systemic erythematous lupus (SLE) [1]. Scleral thickness can be measured with B-scan ultrasonography with high sensitivity and specificity and is the gold standard test for diagnosing posterior scleritis [5]. Optical coherence tomography (OCT) is useful in identifying scleral thickness and can be used to monitor disease activity [6]. Treatment is usually with topical NSAID or corticosteroids; however, they are frequently not effective, and oral NSAID is needed. Systemic steroids are warranted when oral NSAID fail, and steroid-sparing agents such as methotrexate or mycophenolate may be used for persistently active disease or corticosteroid intolerance [6]. In some cases, biologic therapy such as tumor necrotic factor-alpha (TNF) inhibitors or rituximab may be needed [6]. Recurrence occurs in up to one-third of patients and combined treatment by an ophthalmologist and a rheumatologist is recommended [3,6]. Here, we present a case of bilateral posterior scleritis with RA.

## Case presentation

A 46-year-old woman, with a past medical history of bilateral posterior scleritis, was diagnosed by her ophthalmologist with B-scan ultrasound, and RA she presented with bilateral painful eyes, blurry vision, and pain in eye movement. She also complained of left eye swelling. She denied any fever or upper respiratory tract symptoms and no history of recent eye trauma. She had previous episodes of posterior scleritis flares that presented in a similar fashion and had previously been on multiple disease-modifying anti-rheumatic drugs (DMARDs): methotrexate, mycophenolate, rituximab, and infliximab for management but without much success. Most recently, she had been receiving abatacept but had discontinued it two weeks before her current presentation.

On presentation, she was afebrile, and her vital signs were stable. On examination, she had left eyelid swelling, conjunctival erythema, pain with extra-ocular eye movement but no ophthalmoplegia, pupils equal and reactive to light bilaterally, and visual acuity of 20/40 (previously was 20/25). Routine lab workup (complete blood count and basic metabolic panel) was normal, including inflammatory markers: erythrocyte sedimentation rate (ESR) and C-reactive protein (CRP). Attempts made to obtain prior images of actual B-scan ultrasonography were futile as these were performed at an outside facility.

The patient was intolerant to oral steroids and was treated with high-dose intravenous (IV) steroids, as well as IV opioids for pain control in the emergency department. She was subsequently admitted to the general medical floor. She was treated with a high dose of IV methyl-prednisolone, which was tapered over five days, after which she had marked improvements in her ocular symptoms. Of note, the patient’s rheumatologist was consulted and assisted with the treatment regimen. On discharge, she was prescribed prednisolone eye drops and recommended to have a close follow-up with the rheumatologist to restart DMARDs.

**Figure 1:**
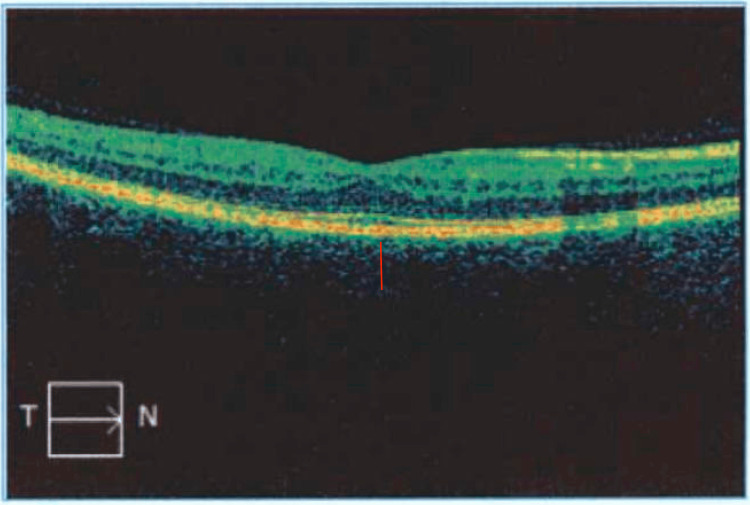
OCT of the right eye (choroidal thickness depicted by the red line) OCT, optical coherence tomography

**Figure 2:**
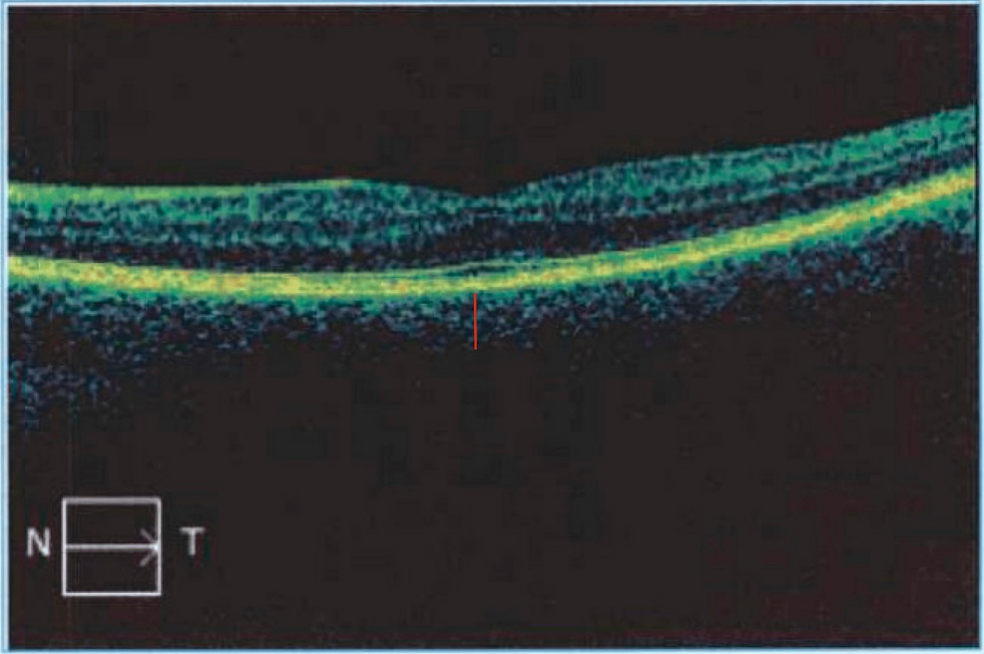
OCT of the left eye (choroidal thickness depicted by the red line) OCT, optical coherence tomography

Eight weeks post treatment of her posterior scleritis flare, she had an ophthalmologic examination with optical coherence tomography (OCT) of both eyes (Figures 1-2), which showed no evidence of retinal or choroidal fluid and stable thickness when compared to prior OCT imaging.

## Discussion

Posterior scleritis is a rare inflammatory condition of the eye. It often manifests as a diagnostic challenge because of its diverse clinical presentation [1]. Females are more likely to develop this disease, and the mean age of presentation is around 45 years [1-3]. It is usually unilateral but rarely can affect both eyes in up to a third of cases [4]. Symptoms of posterior scleritis include eye pain, headache, pain with ocular movements, and loss of vision if not treated timely [1]. Clinical signs are nonspecific and include conjunctival chemosis, hyperemia, and optic nerve swelling [5]. The eye may also be without visible redness if inflammation is only posterior, making the diagnosis even more challenging, and hence a high clinical suspicion is needed [2,6]. Our patient presented with bilateral eye pain and pain in ocular movement with conjunctival erythema. Though she already had a diagnosis of posterior scleritis, her presentation with visible eye redness and swelling makes concurrent anterior scleritis possible. Concurrent anterior scleritis is common in posterior scleritis; in one case series, 59% of patients with posterior scleritis developed anterior scleritis [7].

The condition may masquerade as other ocular pathologies, emphasizing the need for a keen clinical eye to identify its distinct features. Differential diagnoses include acute angle closure glaucoma and orbital/pre-septal cellulitis. Orbital masses such as orbital pseudotumor also known as idiopathic orbital inflammation, rhabdomyosarcoma, leukemia, and neuroblastoma can present in a similar fashion [8,9].

A high index of suspicion is needed to make the diagnosis [2]. About 40-50% of scleritis are associated with systemic diseases, whether noninfectious or infectious [3,8]. The inflammatory condition most commonly associated is RA, but SLE and granulomatosis with polyangiitis (GPA) are commonly linked with posterior scleritis [3,10,11]. Other etiologies of scleritis include infections (namely, varicella zoster virus (VZV) and herpes zoster virus (HZV)), orbital trauma, and medications such as bisphosphonates [8,12]. Other times, the etiology is idiopathic [9]. Our patient had RA.

As internists, primary care providers, and hospitalists, we may be the first healthcare providers to whom patients with this pathology may present. Providers may lack the necessary skills and equipment to make a timely diagnosis of posterior scleritis. This may result in misdiagnosis, wrong treatment, and potentially vision loss in this patient population. Providers must be particularly vigilant when taking care of ocular complaints in patients with autoimmune diseases as they are predisposed to non-infectious posterior scleritis. These patients are usually co-managed with a rheumatologist and an ophthalmologist [6].

Accurate diagnosis hinges on imaging studies to unravel the underlying pathology. A slit lamp exam may show inflammation of the anterior sclera with conjunctival chemosis, vessel dilation, and tortuosity [1]. OCT may show choroidal thickening in active disease and is a useful tool to track disease course, with a reduction of thickening when treated effectively [13].

B-scan ultrasonography is however the cornerstone of imaging in posterior scleritis. It usually shows choroidal and scleral thickening greater than 2.5 mm, as well as fluid in sub-Tenon's space known as the pathognomonic T-sign. In one retrospective case series, the authors proposed a scleral thickness of more than 1.7 mm, which was found to be more sensitive (87.5%) and specific (88.9%) in detecting posterior scleritis, especially in early and mild disease [5]. It is important to note that a negative T-sign does not rule out posterior scleritis, especially when clinical suspicion is high [5]. MRI further compliments the diagnostic arsenal offering a more detailed anatomical outline and is useful when ultrasound is non-conclusive [9]. Collaborative interpretation of these imaging studies can formalize diagnosis and guide subsequent management strategies [3].

Additionally, certain laboratory tests can aid in the diagnosis of this condition, such as a serologic test for rheumatic diseases such as an anti-nuclear antibody (ANA), rheumatoid factor antibody (RF), anti-citrullinated antibody (CCP), and ANCA. For RA, a high RF can clue you into active disease flair, in addition to acute phase reactants, such as CRP and ESR. These markers can also aid in assessing disease activity [10]. Routine labs, such as complete blood count, can show elevated white blood cells, and complete metabolic panel can show signs of liver inflammation and renal insufficiency, which can clue you into underlying systemic diseases such as SLE, autoimmune hepatitis, and small vessel vasculitis such as GPA [1]. 

Essential to management is an accurate diagnosis and a multidisciplinary approach with ophthalmology and rheumatology. Fortunately, our patient was already diagnosed with this rare condition and already established care with rheumatology and ophthalmology. She came in because of a flair for her disease due to recently stopping her immunosuppressant therapy. This case report will further explore the treatment landscape of posterior scleritis. The use of topical corticosteroids and oral non-steroidal anti-inflammatory drugs are first-line therapies [7,8]. These first-line therapies are usually effective in young patients with unilateral scleritis and those without associated systemic diseases. A substantial number of patients with mild-to-moderate disease will need alternative treatment such as systemic steroids [7,8].

Oral corticosteroids remain the foundation of short-term management of scleritis, and the usual starting dose of prednisolone is 1 mg/kg/day with weekly tapering depending on the clinical response [6,7]. Pulse IV corticosteroid use is imperative, especially in vision-threatening cases where prompt control of inflammation is necessary [7]. In our patient, her vision was significantly impaired, warranting IV corticosteroid use, with improvement in symptoms. Additionally, she was intolerant to oral steroids. Posterior scleritis may recur frequently, and long-term immunosuppressant therapy is usually warranted [3,8]. There are challenges posed by long-term corticosteroids due to their many side effects. The addition of steroid-sparing agents is indicated in failure or inadequate response to steroids, recurrent disease on more than 10 mg/day of oral corticosteroid, and serious side effects of steroids [7]. Our patient had a trial of multiple DMARDs with the recurrence of her disease, further highlighting the recurrent nature of this disease. 

The use of methotrexate was the most selected first-choice steroid-sparing therapy in a study of treatment preferences in the management of scleritis [7]. Some factors that influenced this were its ease of weekly administration, patient tolerance, and flexible dosing. It is however associated with a faster time to relapse [11]. Other steroid-sparing agents to consider are azathioprine and mycophenolate for their tolerability and more rapid steroid-sparing effect when compared to methotrexate [7]. Cyclophosphamide is another agent used, but primarily in patients with systemic vasculitis such as GPA [6,8]. A limiting factor in its use is its side effect profile with no increase in efficacy when compared to other immunosuppressant therapies [7].

Biologic therapy has become very useful in the management of posterior scleritis due to its targeted approach of modulating the inflammatory cascade. In one study, TNF-alpha inhibitors were the first-choice biologic agent used to manage scleritis among rheumatologists and uveitis specialists [7]. TNF-alpha inhibitors such infliximab and adalimumab are great options in this arsenal as they have been shown to be effective in the management of treatment-resistant scleritis [7]. Golimumab has been shown to be effective in the treatment of severe refractory posterior scleritis on a case-report basis [14]. Rituximab is another first-line biologic medication that has been shown to be well-tolerated and effective in controlling scleral inflammation in refractory scleritis, but the recurrence rate is high requiring repeated infusions [7,8,14]. Newer biologic agents such as tocilizumab an interleukin-6 inhibitor have shown promise in the treatment of scleritis, especially with severe systemic rheumatoid vasculitis with multiple extraarticular diseases and scleritis [7]. Janus kinase inhibitors such as tofacitinib are also effective in the treatment of scleritis [7]. Abatacept is a T-cell costimulator modulator that inhibits T-cell activation, thereby disrupting the immune-mediated inflammatory response to the sclera [15]. Based on data from a case report, its use has been shown to significantly induce remission in posterior scleritis [16].

Our patient had multiple relapses despite undergoing treatment previously with methotrexate, mycophenolate, rituximab, and infliximab. Most recently, a flair occurred while being off abatacept for two weeks. This case underscores the potential efficacy of biologics in managing refractory cases of posterior scleritis. However, the lack of robust evidence from well-designed and adequately powered studies regarding the use of biologics emphasizes the need for further research to establish management strategies for posterior scleritis and scleritis overall.

## Conclusions

Posterior scleritis is a rare yet serious inflammatory condition affecting the sclera, which can pose a significant threat to vision if not treated promptly. Bilateral disease is even rarer, as seen in our patient, and can be associated with concurrent anterior scleritis. As providers maintaining a high index of suspicion is crucial to recognize this condition early to prevent vision loss. A multidisciplinary approach with rheumatology and ophthalmology is needed to tailor specific treatment strategies. Though treatment is challenging, when conventional therapies prove inadequate, the use of biologics becomes a valuable consideration, especially with relapsing disease, as seen in our patient. While biologic therapy holds promise in the treatment of posterior scleritis, larger studies are warranted to further expand on evidence-based treatment guidelines.
